# Fluorescence lifetime imaging of endogenous biomarker of oxidative stress

**DOI:** 10.1038/srep09848

**Published:** 2015-05-20

**Authors:** Rupsa Datta, Alba Alfonso-García, Rachel Cinco, Enrico Gratton

**Affiliations:** 1 Laboratory of Fluorescence Dynamics, Department of Biomedical Engineering, University of California, Irvine; 2 Department of Biomedical Engineering, University of California, Irvine; 3 Department of Developmental & Cell Biology, University of California, Irvine

## Abstract

Presence of reactive oxygen species (ROS) in excess of normal physiological level results in oxidative stress. This can lead to a range of pathological conditions including inflammation, diabetes mellitus, cancer, cardiovascular and neurodegenerative disease. Biomarkers of oxidative stress play an important role in understanding the pathogenesis and treatment of these diseases. A number of fluorescent biomarkers exist. However, a non-invasive and label-free identification technique would be advantageous for in vivo measurements. In this work we establish a spectroscopic method to identify oxidative stress in cells and tissues by fluorescence lifetime imaging (FLIM). We identified an autofluorescent, endogenous species with a characteristic fluorescent lifetime distribution as a probe for oxidative stress. To corroborate our hypothesis that these species are products of lipid oxidation by ROS, we correlate the spectroscopic signals arising from lipid droplets by combining FLIM with THG and CARS microscopy which are established techniques for selective lipid body imaging. Further, we performed spontaneous Raman spectral analysis at single points of the sample which provided molecular vibration information characteristics of lipid droplets.

Reactive oxygen species (ROS) are intrinsic free radicals produced as a result of normal cellular metabolism. ROS concentration at moderate level plays a role in signaling pathways of physiological processes and in maintaining redox homeostasis^1^,^2^,^3^. However, increased concentration of ROS causes oxidative stress. This is detrimental to the cellular components because of several biochemical processes including lipid peroxidation and proteins and DNA damage^3^. Modifications of these biomolecules could ultimately lead to a number of human diseases such as inflammation, diabetes mellitus, atherosclerosis, cancer, and neurodegenerative disease^4^,^5^,^6^,^7^,^8^,^9^,^10^. Therefore, biomarkers of oxidative stress play an important role in understanding the pathogenesis and treatment of these diseases.

Detecting ROS itself is a direct measure for identifying the presence of oxidative stress. ROS-specific fluorescent indicators are available commercially. However, the use of these indicators requires administration of a foreign material to the physiological environment. Instability of ROS molecules and further perturbation of biological systems by the current invasive ROS detection techniques make this a difficult task. Indirect techniques for detecting ROS utilize the more stable ROS oxidation products. These identify damage to biomolecules by ROS or quantify levels of antioxidants or redox molecules. In this work, we show label-free detection of oxidative stress by fluorescence lifetime measurement of intrinsic fluorescent species using multiphoton fluorescence microscopy. These species with granular appearance co-localize with lipid droplets. We hypothesize that the identified species are products of lipid oxidation by ROS. A similar preliminary observation was reported previously in human embryonic stem cells^11^. The identified endogenous biomarker unfolds opportunities of performing non-invasive measurements of oxidative stress in vivo.

Multiphoton fluorescence microscopy (MPM) has been employed previously to perform label free fluorescence lifetime imaging (FLIM) of intrinsic fluorophores like reduced nicotinamide adenine dinucleotide (NADH), collagen, retinol, and retinoic acid^11^,^12^. The main advantages of MPM are reduced phototoxicity and higher penetration depth, needed for in vivo measurements especially in tissue samples. Endogenous fluorophores enable non-invasive imaging of biological samples, minimizing the perturbation of normal physiological conditions. For example, autofluorescent metabolic coenzymes flavin adenine dinucleotide (FAD) and NADH are frequently employed as probes of metabolism for label-free imaging^13^,^14^. For analyzing the fluorescent decay in FLIM images, we employed the phasor approach. This method simplifies and speeds up the analysis because it works on the raw data without the need to perform a fit of the fluorescence decay at each point of an image^15^. The method does not require a priori knowledge of the fluorescence lifetime components in the imaged sample and gives instantaneous results. Briefly, the data from each pixel of the image are subjected to a Fourier transformation to obtain the corresponding phasor as previously described^11^,^15^. In the phasor approach we can identify separate clusters of species with different lifetimes. The lifetime information shown in the phasor plot can be mapped back to the image to resolve the spatial location of these species.

To validate the concurrence of lipid droplets with the identified oxidative stress biomarkers, we combined the FLIM approach with two coherent nonlinear microscopy techniques: third harmonic generation (THG) imaging microscopy and coherent anti-Stokes Raman scattering (CARS) microscopy. It is known that a strong THG signal is generated at the interface between media with difference in third order nonlinear susceptibility, refractive index and dispersion. In particular it has been shown that the interface between a lipid droplet and its surrounding produces a strong THG contrast^16^. Hence, the technique can be employed to selectively identify lipid bodies in biological samples. CARS is also a label-free technique used for imaging neutral lipid droplets. The contrast of the CARS signal in the lipid droplets arises from the Raman response of the abundant C-H bonds in the lipid molecules^17^. Thus, laser scanning CARS microscopy is applied to visualize lipid droplets in cells and tissues. Both of these techniques have the advantage of being label-free and non-invasive while they can still be correlated to the results of FLIM imaging.

To further investigate the chemical nature of the observed species, we performed classical Raman spectral analysis. Raman spectroscopy has the advantages of providing high molecular selectivity^18^, and non-invasiveness, especially compared to techniques like mass spectroscopy. We employed a confocal Raman microscope where we could select specific locations on the biological sample, and acquire Raman spectra from these areas. For identifying lipids on our samples, we looked at the fingerprint region and the C-H stretching vibration region. Characteristic spectra of lipid droplets have previously been identified in these Raman bands^19^,^20^.

In this work, we establish a non-invasive, label-free MPM method to identify a biomarker of oxidative stress. We found the identified endogenous fluorescent species to have a characteristic fluorescence lifetime distribution on the phasor plot. MPM imaging provided high resolution imaging and by phasor analysis of FLIM, this biomarker could be easily identified in live cells and ex-vivo tissues and correlated to lipid droplet locations. We hypothesize that the fluorescent species is a product of lipid oxidation by ROS. We show co-localization of the spectroscopic signals to lipid droplets by combining FLIM with THG and CARS microscopy which are techniques employed for selective lipid body imaging. Raman spectral analysis on the regions with this characteristic lifetime provided additional evidence of molecular vibrations arising from oxidized lipids.

## Results

### Identification of a unique long lifetime species in white adipose tissue by FLIM

**Figure 1:**
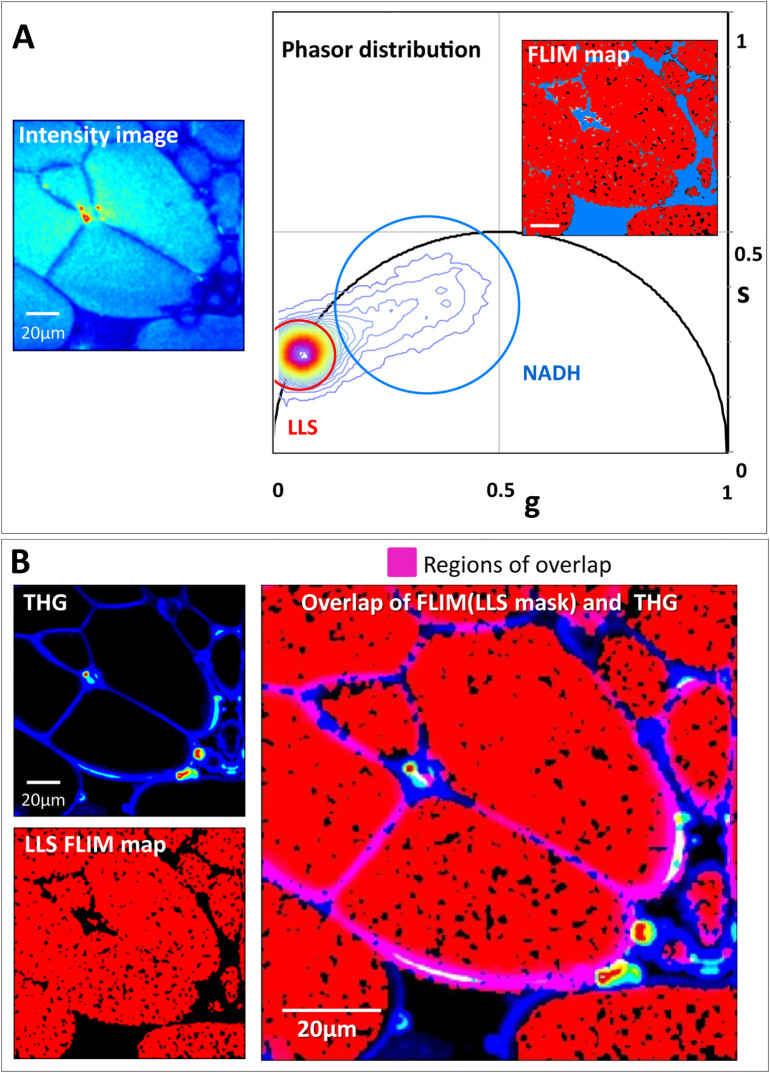
Unique fluorescence long lifetime signature (LLS) in white adipose tissue and correlation with THG image. (A). Left panel is average fluorescence intensity image of white adipose tissue excited at 740 nm. Right panel is the corresponding phasor distribution. The red circular cursor chooses the long lifetime distribution cluster while blue cursor chooses the NADH phasor distribution. The inset image is the FLIM pseudo-color map with red and blue regions corresponding to the two identified phasor clusters selected with red and blue cursors respectively. Scale bar is 20 µm. (B). Top left panel is the THG intensity image with the sample excited at 1038 nm. Bottom left panel is the FLIM map of long lifetime species (LLS). Right panel is the composite image of LLS FLIM map and THG image.

We performed fluorescence lifetime imaging measurements on freshly excised perigonadal white adipose tissue (WAT) using endogenous autofluorescence. Performing the phasor analysis of the acquired FLIM data, we identified a long lifetime distribution cluster in the phasor plot (Fig. 1A, encircled in red cursor) of 7.8 ns. This distribution of the long lifetime species (LLS) has a distinct position on the phasor plot separate from the NADH-FLIM signature (Fig. 1A, encircled in blue cursor). The LLS and NADH-FLIM phasor clusters are mapped back onto the intensity image (Fig. 1A, right panel inset image). The tissue regions exhibiting the long lifetime component are colored red. These pixels correspond to the areas within the large lipid droplets present in the adipocytes. The NADH-FLIM cluster is chosen by the blue cursor on the phasor plot, and this selection corresponds to regions surrounding the lipid droplet: adipocyte cytoplasm, nuclei, and the extracellular regions. To further ascertain that the pixels selected by the red cursor are associated to lipids, we performed THG imaging on the same region. We found a strong THG signal arising from the periphery of lipid droplets (Fig. 1B left top panel). The LLS FLIM map overlaid on the THG image shows that the periphery of regions with long fluorescence lifetime have strong THG signal (Fig. 1B right panel, regions of overlap in pink). This is consistent with our hypothesis that we are observing lipid droplet associated autofluorescence. Based on FLIM and THG results, we manually drew, on the fluorescence intensity image, a region of interest (ROI) in two different lipid droplets (Supplementary Fig. S1). The phasor distribution associated to these ROIs shows a unique component on the universal circle which corresponds to a single exponential lifetime at 7.8 ns.

**Figure 2:**
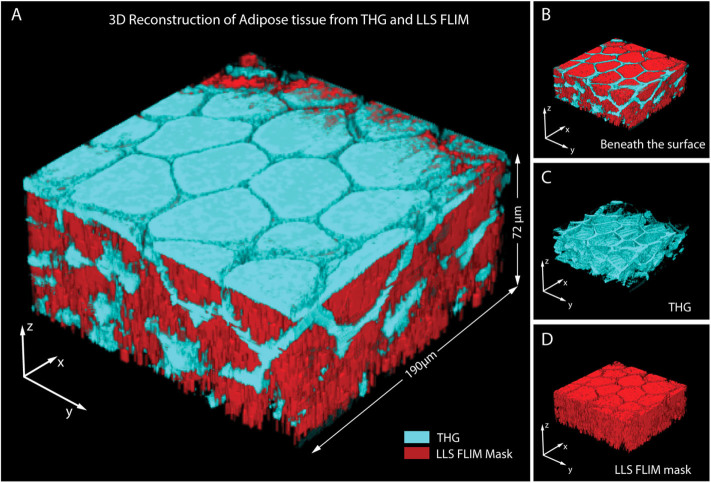
Simultaneous THG and FLIM in WAT reconstructed from 25 z – slices at every 3 µM of the tissue. (A). 3D reconstructed THG signal from white adipose tissue in cyan overlapped with long lifetime FLIM map in red (B). THG and FLIM from the same tissue sample reconstructed from below the top surface (C). 3D reconstructed only THG signal from below the top surface (D). Corresponding 3D FLIM map of long lifetime species in the tissue.

We acquired FLIM and THG of 25 z-slices at every 3 µm of the visceral white adipose tissue from surface to a depth of 72 µm. Figure 2A is the 3-D reconstruction of THG (cyan) and LLS FLIM map (in red), and Fig. 2B shows the 3-D reconstruction beneath the surface of the tissue with both signals merged. Figure 2C and 2D are the THG and LLS FLIM signals respectively.

**Figure 3:**
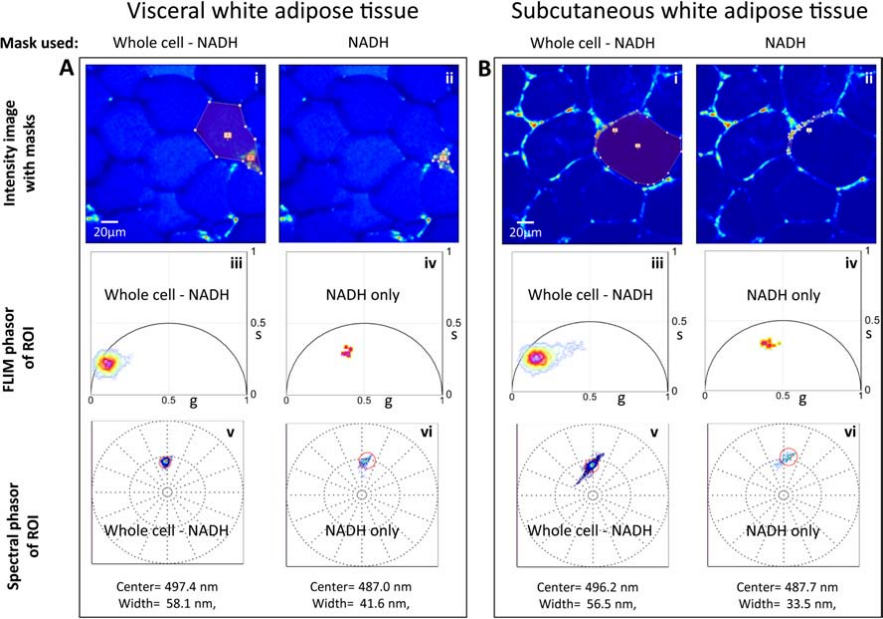
FLIM and spectral phasor distribution of visceral and subcutaneous white adipose tissue. (A). Top panel are fluorescence intensity images of visceral white adipose tissue with the ROI masks [Whole cell – NADH ] (A i) and [NADH] (A ii). Middle panel are FLIM phasor distribution from the masks. Aiii shows FLIM phasor distribution from mask in (Ai) while (A iv) shows FLIM phasor distribution from mask in (Aii). Bottom panels are spectral phasor distribution from the masks. (Av) shows FLIM phasor distribution from mask in (Ai) and (A vi) shows FLIM phasor distribution from mask in (Aii). (B). Top panel are fluorescence intensity images of visceral white adipose tissue with the ROI masks [Whole cell – NADH] (B i) and [NADH] (B ii). Middle panel are FLIM phasor distribution from the masks. (B iii) shows FLIM phasor distribution from mask in (Bi) while (B iv) shows FLIM phasor distribution from mask in (Bii). Bottom panels are spectral phasor distribution from the masks. (Bv) shows FLIM phasor distribution from mask in (Bi) and (Bvi) shows FLIM phasor distribution from mask in (Bii).

To investigate the differences of the long lifetime species in visceral versus subcutaneous WAT, we performed FLIM on visceral fat depot (perigonadal WAT) and subcutaneous fat depot (flank area) of the same animal. We also collected spectral emission data from the same tissue area and analyzed the emission characteristics using the spectral phasor approach method described previously^21^. Fig. 3A shows the FLIM phasor and spectral phasor analysis in the field of view for the two kinds of WATs. Separate masks were applied manually to select the whole cell (Mask 1) and the region with NADH FLIM signature (Mask 2) within the selected cell. For generating Mask 2 we were guided by the FLIM phasor distribution where the decay of NADH can be distinguished. The Boolean XOR operation was performed between Mask 1 and Mask 2 to obtain the resulting *Whole cell–NADH* mask. The FLIM phasor distribution (Fig. 3A, iii, Visceral WAT and Fig. 3B, iii, Subcutaneous WAT) and the spectral distribution (Fig. 3A, v, Visceral WAT and Fig. 3B, v, Subcutaneous WAT) were mapped back onto the phasor plot after application of *Whole cell–NADH* mask on the imaged area. The FLIM phasor distribution (Fig. 3A iv, Visceral WAT and Fig. 3B iv, Subcutaneous WAT) and the spectral phasor distribution (Fig. 3A vi, Visceral WAT and Fig. 3B vi, Subcutaneous WAT) were mapped back onto the phasor plot, after application of the *NADH* mask. The spectral phasor method was employed together with lifetime measurements to determine the characteristic emission average spectrum of the areas that correspond to long lifetime species (LLS) and those with NADH FLIM signature. The spectral phasor distribution of the LLS was centered on 497.4 nm for visceral WAT and 496.2 nm for subcutaneous WAT. NADH spectral phasor distribution was centered at 487 nm for both the WATs. Supplementary Fig. S2 shows the FLIM and spectral phasor distribution of the complete imaged areas of visceral and subcutaneous WAT.

### FLIM phasor signature of long lifetime species

**Figure 4:**
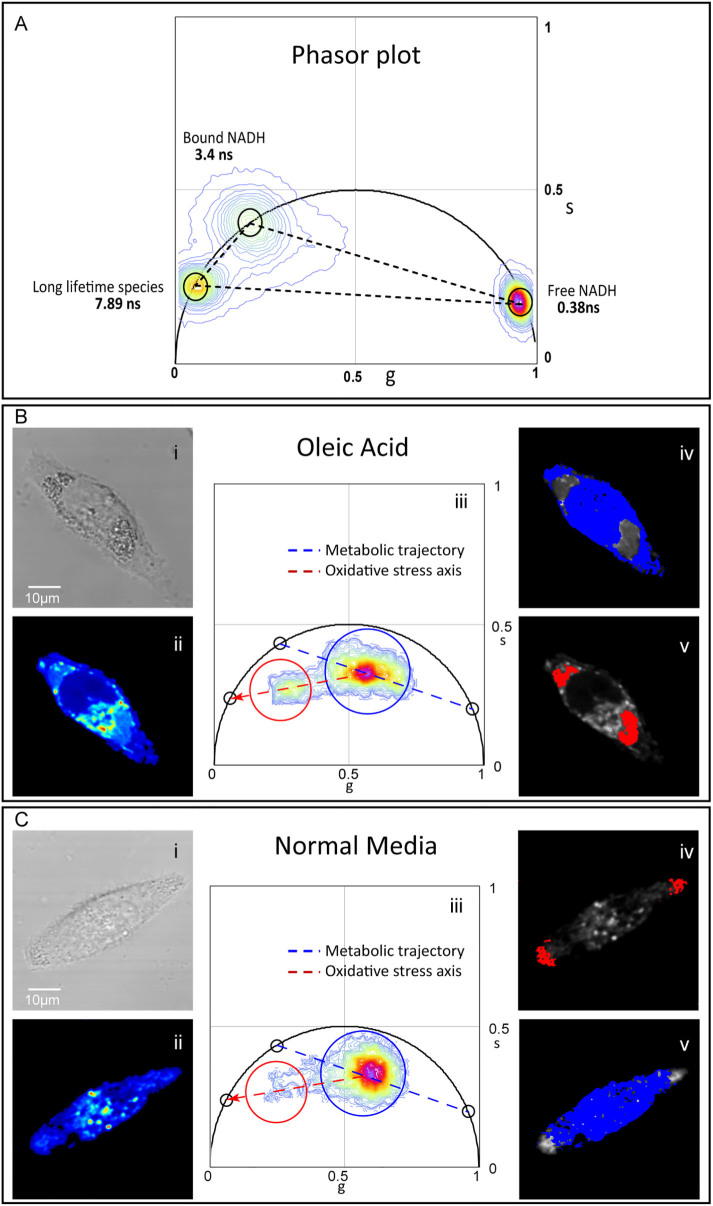
Unique LLS FLIM signature and a new oxidative stress axis on phasor plot. (A). Phasor plot showing triangle formed by FLIM fingerprints of free 2.5 µM NADH in solution, NADH bound to 0.75 U/ml Lactate dehydrogenase (LDH) enzyme and unique LLS from lipid droplets in perigonadal WAT of female mouse. From the law of phasor addition, a system containing mixtures of these three species will fall within the triangle joining the three phasors. (B). FLIM of oleic acid fed HeLa cell. Bi is the transmission image and Bii is the corresponding fluorescence intensity image with the sample excited with 740 nm and emission collected using bandpass filter 480/80 nm. Biii is the resulting phasor distribution from the sample with blue cursor selecting the NADH phasor distribution and red cursor choosing the LLS cluster. Blue dotted line is the metabolic trajectory while red dotted line is the oxidative stress axis. Biv is the NADH map where pixels with lifetime within the blue cursor in Biii are colored blue. Bv is the LLS map where pixels with lifetime within the red cursor in Biii are colored red. (C). FLIM of HeLa cell in normal media but exhibiting LLS. Ci is the transmission image while Cii is the corresponding fluorescence intensity image using same excitation and emission as B. Ciii is the resulting phasor distribution from the sample. The blue and red cursors as well as the metabolic trajectory and the oxidative stress axis are kept at same positions as Biii. Civ is the corresponding NADH map while Cv is the LLS map.

In the phasor approach to FLIM, according to the vector law of phasor addition, if a pixel contains a mixture of two molecular species, the corresponding phasor will be distributed along a line joining phasors of the two pure species^15^. Here we have a system of three molecular species, namely free NADH, protein bound NADH, and oxidized lipid associated fluorescent species (LLS). The line joining the free and protein bound NADH, which is from 0.4 ns to 3.4 ns in the phasor plot, has been previously named the “metabolic trajectory”^11^,^12^ in the phasor plot. From Fig. 1 and Fig. 2, the FLIM signature of pure LLS can be established to be 7.8 ns. The phasor of a pixel in the image containing a mixture of the three species will lie inside the triangle whose vertices are formed by the phasors of the three pure species on the phasor plot (Fig. 4A). For the NADH lifetime signature, FLIM of pure free NADH and NADH bound to lactate dehydrogenase was used to locate the extremes of the bound and free NADH trajectory. The distribution of LLS was obtained from the WAT in Fig. 1.

### Long lifetime species in HeLa cells treated with oleic acid

For characterizing the biochemical origin the LLS associated with lipid and oxidative stress, we treated HeLa cells with oleic acid to stimulate lipid droplet formation in the cells. Oleic acid supplementation often results in increased neutral lipid accumulation in form of lipid droplets^22^,^23^. Additionally, it has been reported that oleic acid increases ROS generation and oxidative stress^24^,^25^. As a control, we cultured HeLa cells in normal media as well as lipoprotein deprived serum (LPDS) media, which are not expected to generate elevated numbers of lipid droplets or cause additional stress. In the FLIM phasor distribution of oleic acid fed HeLa cells (Fig. 4B, iii), we could identify populations with NADH (blue cursor) and LLS (red cursor) FLIM signatures. NADH phasor distribution in the imaged cell falls along the metabolic trajectory shown by a blue dotted line in Fig. 4B, iii. Using the phasor approach to FLIM, we mapped these populations back to the image to visualize the regions of identified lifetime clusters on the phasor plot. The NADH phasor distribution cluster corresponds to the nucleus and cytoplasm of the cell (Fig. 4B, iv). The LLS phasor distribution falls along the line joining the center of NADH distribution and the pure LLS FLIM signature on the universal circle. This oxidative stress trajectory shown by a red dotted line in Fig. 4B, iii lies within the triangle formed by the phasors of free NADH, protein bound NADH, and LLS on the phasor plot, as shown in Fig. 4A. When mapped back onto the image, regions with long lifetime distribution, chosen by the red cursor, correspond to the intracellular lipid droplets of the oleic acid treated HeLa cells (Fig. 4B, v).

A small percentage of control HeLa cells cultured in normal media also displayed the LLS phasor distribution (Fig. 4C). However, when the long lifetime species is present, it usually appears in lipid droplets near the cellular membrane.

**Figure 5:**
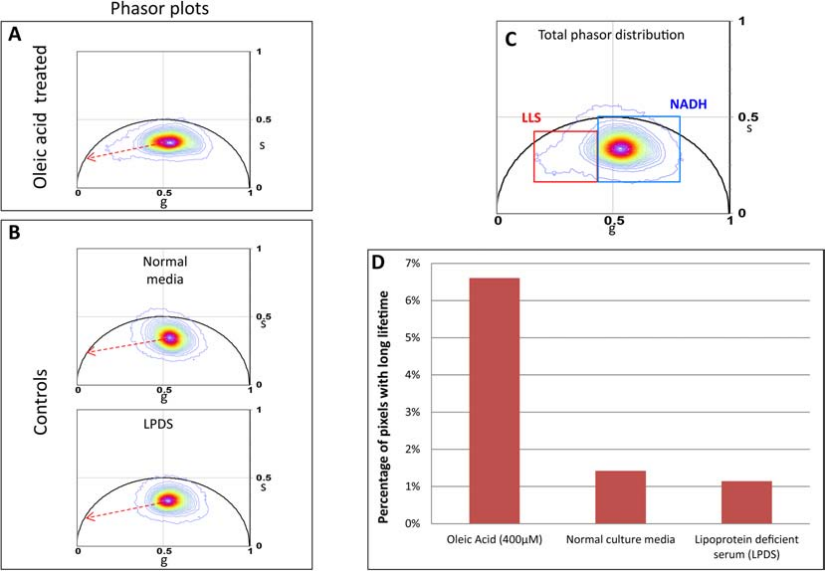
Increase in areas with LLS in oleic acid treated HeLa cells. (A). Phasor distribution of HeLa cells treated with 400 *μ*M oleic acid for 24 hours. Red dotted line shows the oxidative stress axis. (B). Phasor distribution of HeLa cells in normal media (top panel) and in lipoprotein deficient serum, LPDS (bottom panel). Red dotted line shows the oxidative stress axis. (C). Binary division of the phasor distribution with LLS window (red square) selecting pixels with longer lifetime and NADH window (blue square) selecting the shorter lifetime distribution. (D). Bar graphs showing percentage of pixels within lifetime in the LLS window in the three groups.

In Fig. 5 we show FLIM phasor distribution of the three groups of HeLa cells (oleic acid treated, normal media, and LPDS). For each group, FLIM analysis was performed on 12 areas. This included 55 oleic acid fed cells, 58 cells cultured in normal media, and 54 cells in LPDS media. Comparing the individual phasor distribution of the three groups, the LLS population along the oxidative stress axis is found to be markedly pronounced in the oleic acid fed group (Fig. 5A and Fig. 5B), while it is negligible in the control groups.

For statistical analysis, the phasor distributions of all three groups were divided into two windows, NADH (blue square) and LLS (red square) as shown in Fig. 5C. We calculated the fraction of pixels in all the acquired images with phasors in the NADH window and the LLS window. These values were normalized to the total number of pixels with phasors in the two windows and converted into percentage. The percentage of pixels in the LLS window in the three groups are plotted in Fig. 5D. The plot shows a 6-fold increase in the lipid droplet associated LLS in HeLa cells treated with oleic acid compared to the control cells in normal media and LPDS media.

### Use of non-linear label free microscopy techniques to determine the origin of the autofluorescence signal

**Figure 6:**
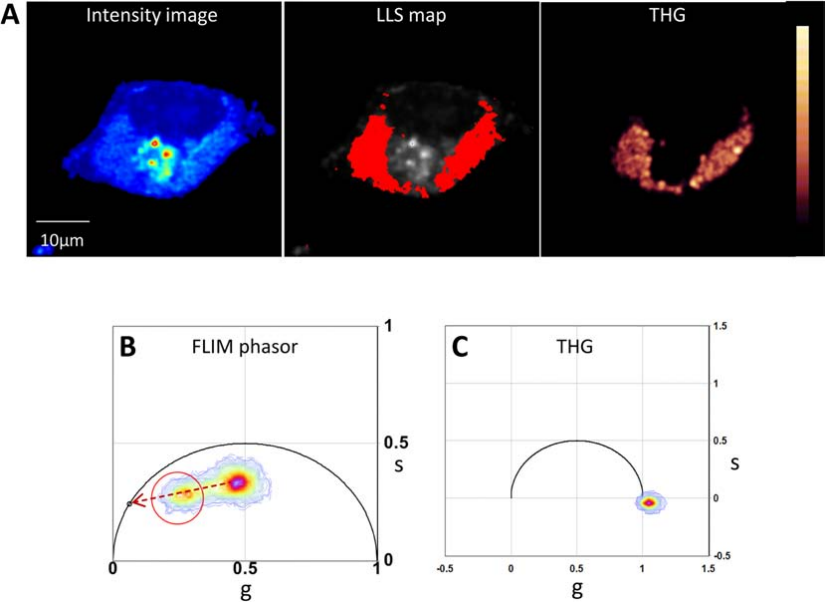
Sequential FLIM and THG imaging of oleic acid treated fixed HeLa cell (A). Left panel is average fluorescence intensity image of treated HeLa cell excited at 740 nm. Middle panel is the FLIM map of long lifetime phasor cluster selected by red cursor in the phasor plot (B). Right panel is the THG signal from the same sample excited at 1038 nm. (B). FLIM phasor plot with red cursor selecting the long lifetime cluster. (C). THG phasor plot.

To further elaborate on our hypothesis that the long lifetime component arises from oxidized lipid associated autofluorescence, we employed THG and CARS imaging along with FLIM of oleic acid fed HeLA cells. Both these techniques offer additional contrasts for observing lipid structures. Figure 6 shows the results of simultaneous FLIM and THG imaging of oleic acid treated fixed HeLa cell. In the phasor plot we identified the LLS cluster along the oxidative stress axis (red cursor in the phasor plot, Fig. 6B) and mapped them on the FLIM image (Fig. 6A middle panel). The area selected by the red cursor falls within the THG signal (Fig. 6A right panel) arising from lipid droplets. Supplementary Fig. S3 demonstrates similar LLS lifetime distribution of fixed and live oleic acid treated HeLa cells (n = 3) thus indicating that fixation of the cells does not affect the LLS lifetime.

**Figure 7:**
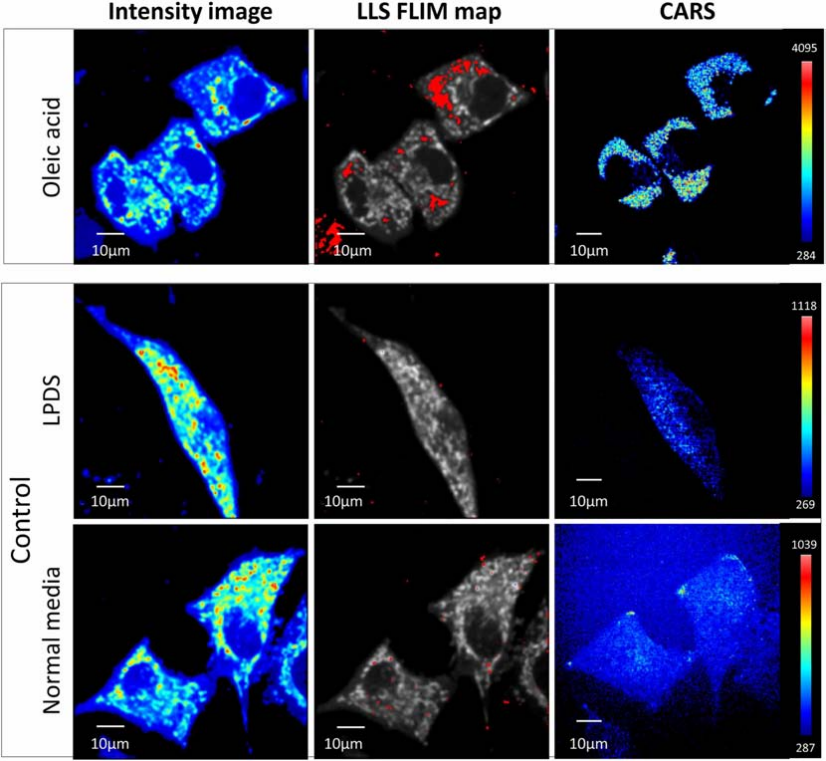
FLIM and CARS imaging of oleic acid treated HeLa cells. Top panel is fluorescence intensity image, LLS FLIM map in red and CARS image of oleic acid fed HeLa cells. Bottom panel is fluorescence intensity image , LLS FLIM map in red and CARS image of the control groups of HeLa cells in LPDS media (top row) and in normal media (bottom row).

Further proof for the oxidized lipid origin of LLS was obtained from combined FLIM and CARS imaging. Once again we performed imaging on three groups of HeLa cells : oleic acid-treated, LPDS, and normal media (Fig. 7). The group treated with oleic acid had much stronger CARS signal, revealing abundant and larger lipid droplets compared to the other two groups. This also correlates with increased areas of oxidized lipid associated autofluorescence in the oleic acid fed group (Fig. 7, middle column). However, it should be noted that not all lipid droplets, unveiled by CARS signal, exhibit the LLS signature. Supplementary Fig. S4 shows additional LLS FLIM map and CARS images of the three groups. Interestingly, comparing the FLIM and CARS signals, we noticed few cells (white arrow) with LLS signature that are absent in the CARS images. These cells with strong oxidized lipid signal might have undergone apoptosis precisely due to oxidative stress, and were washed off when the media was changed before CARS imaging.

### Chemical analysis by Raman spectroscopy

**Figure 8:**
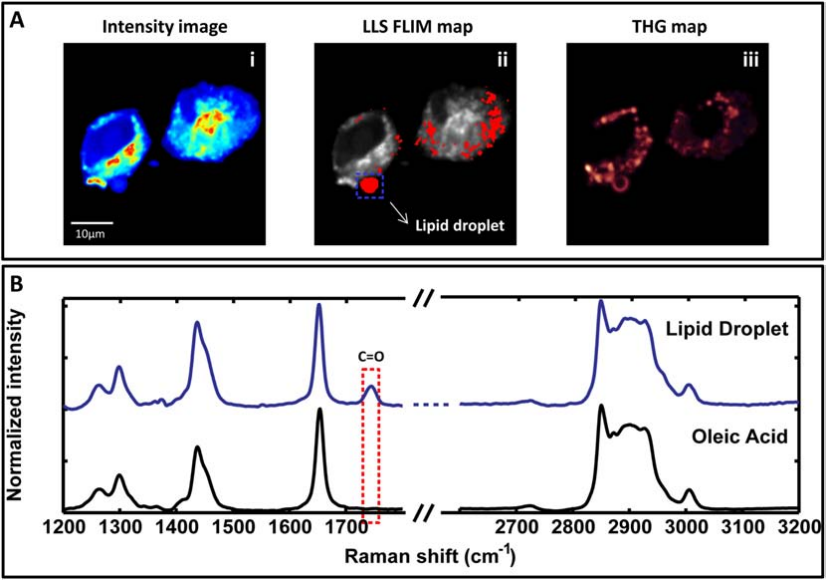
Chemical characterization of LLS by Raman spectroscopy. (A). Fluorescence intensity image (Ai) of fixed oleic acid fed HeLa cells. Aii is the LLS FLIM map in red. Dotted blue box encloses the lipid droplet with LLS signal from where the Raman spectrum was acquired. Aiii is the THG image from the same area. (B). Blue curve is the Raman spectra from the lipid droplet of interest (marked by a blue square in Aii). Black curve is the Raman spectra from 90% pure oleic acid. Red dotted box highlights the additional peak observed in the Raman spectra from biological sample which is not a feature of oleic acid

For chemical characterization of the oxidized lipid associated species with autofluorescence signal, we obtained Raman spectra at regions of oleic acid loaded HeLa cells that displayed the unique LLS FLIM signature. In Fig. 8A, ii, the lipid droplet from where Raman spectra was obtained has been indicated by a blue square. This region also had a strong THG signal (Fig. 8A, iii). The blue curve in Fig. 8B shows the Raman spectra of a lipid droplet in the fingerprint (1200–1800 cm^−1^) and the CH stretching (2700–3200 cm^−1^) regions. We also obtained Raman spectra of 90% pure oleic acid (Fig. 8B black curve). The Raman spectra in the CH stretching regions acquired from the LLS containing lipid droplets show the typical vibrational bands of pure oleic acid. There is an additional peak in the fingerprint region at 1746 cm^−1^, which is assigned to the C = O stretching mode of ester bonds. This bond is formed upon esterification of the fatty acid into neutral triglycerides, which constitute the major component of lipid droplets. Supplementary Fig. S5 shows similar Raman spectra obtained from lipid droplets in two different oleic acid fed fixed HeLa cells with LLS signatures and THG signal. For this analysis, Raman spectra were normalized by sections. The fingerprint band data were normalized to the 1646 cm^−1^ Raman band, whereas the CH stretching band data were normalized to the 2850 cm^−1^ Raman band.

## Discussion

In this work we identified a fluorescent species with unique long lifetime properties (around 7.8 ns) which is distinct from the common NADH lifetime in cells (1–2 ns). We present results showing autofluorescent long lifetime species (LLS) linked to products of lipid oxidation by ROS, and hence potential biomarker for oxidative stress. Lipids, per se, are non-fluorescent, however, oxidized lipids can be^12^. We found that most of the fluorescence arises from lipid droplets with granular structure. This long lifetime distribution found in cells represents a species different from free/bound NADH. In this work we grouped both nuclear and cytoplasmic NADH as a single distribution, although phasor analysis can separate NADH from the two sub cellular regions. To identify the source of fluorescence in the LLS, we performed FLIM imaging of freshly excised visceral and subcutaneous WAT from old female mice. We found the LLS phasor distribution from the adipocyte lipid droplets to fall on the universal circle at 7.8 ns. This indicates the existence of a pure chemical species in the lipid droplet, as it is known that lifetime distribution of pure species with single exponential decay would lie on the universal circle^15^. This also confirms the existence of a species separate from NADH in our sample. NADH distribution in biological samples generally falls on the metabolic trajectory, the line joining position of pure free NADH (0.4 ns) and pure protein bound NADH (3.4 ns)^11^. This metabolic trajectory and the LLS signature on the universal circle form a triangle on the phasor plot. From phasor algebra, a pixel in the image where LLS can coexist with the NADH distribution will have a position inside this triangle. Hence using the phasor approach, it was possible to distinguish these separate populations and map them back to the images to reveal their spatial locations.

To investigate the unique LLS in cells and their association with lipid droplets, we used HeLa cells fed with oleic acid to stimulate the formation of lipid droplets, and we observed the LLS signal arising from the lipid droplets. In these samples, the linear combination of the long lifetime components due to oxidized lipids species and NADH autofluorescence give rise to a separate, easily identifiable cluster of phasors. This distribution cluster falls along the line joining the center of NADH distribution and LLS FLIM signature (7.8 ns) on the universal circle. We propose this line as the new oxidative stress axis on the phasor plot. Mapping the lifetime phasors back onto the images, we observed the lipid droplet with LLS were present in the cytoplasm of the HeLa cells. In rare occasions the LLS were also observed in a few cells grown in normal media. Interestingly, these LLS containing lipid droplets were mostly localized towards the membrane of the cells. We explored the fluorescence emission characteristics of the LLS by employing spectral phasors^21^. By comparing FLIM phasor and spectral phasor analysis of both visceral and subcutaneous WAT, we found the regions with the LLS FLIM signature to have distinct emission spectral properties than NADH. The LLS spectral phasor distribution was centered on 497.4 nm for visceral WAT and 496.2 nm for subcutaneous WAT. These were separable from NADH spectral phasor distribution centered on 487 nm for both tissues.

To identify the cellular location of the intrinsic fluorescent species, we imaged tissue and cells using THG microscopy along with FLIM. THG signal arises from the interface between the lipid droplets and their surroundings thus revealing the spatial location of the droplets. Coupling the two modalities of label-free imaging, we verified the co-localization of LLS within the lipid droplets of adipocytes in visceral WAT. A 3D reconstruction of FLIM and THG images through 72 µm depth of visceral WAT exhibited LLS signal throughout the lipid droplets of the adipocytes surrounded by strong THG signal from the periphery of the droplets. This is where the FLIM and THG signals overlapped. Simultaneous FLIM and THG from oleic acid fed HeLa cells displayed the same results.

Additionally, to test the chemical origin of the fluorescence signal we performed FLIM on oleic acid fed HeLa cells followed by CARS imaging of the same cells. This was possible due to the label-free and non-invasive nature of both imaging techniques. CARS images revealed a large amount of lipid droplets in the samples, supporting the increase in LLS phasor distribution along the oxidative stress axis. These results once more substantiate our hypothesis of association of LLS with lipid oxidation products. Interestingly, not all lipid droplets detected both by CARS and THG have the autofluorescence long lifetime signature.

Confocal Raman spectroscopy allows non-invasive chemical analysis of biological samples. We employed this technique to analyze the locations where the long lifetime components were detected. Fixed HeLa cells with oleic acid induced lipid droplet were imaged by FLIM, and Raman spectra were acquired subsequently from granules with LLS FLIM signature. The Raman spectra of the regions with LLS displayed the Raman signatures indicative of esterified oleic acid, characteristic from triglycerides in the lipid droplets^19^,^20^,^26^,^27^.

Lipid droplet associated autofluorescence has been identified previously. These include lipofuscin granules and retinosomes. Lipofuscin granules are found in human retinal pigment epithelial (RPE) cells, fibroblasts and other types of cells and have also been reported as oxidative stress marker^28^,^29^. Stringari et al reported existence of long lifetime species (about 8 ns) in human embryonic stem cells and co-localized these lipid granules with 4,4-difluoro-1,3,5,7,8-pentamethyl-4-bora-3a,4a-diaza-s-indacene (BODIPY493/503), which is a stain for neutral lipids^11^. They established that the autofluorescent species with long lifetime were not associated to lipofuscin, which has a much shorter lifetime (See Supplementary Fig. 6)^30^. This ascertains the LLS reported in this study are not related to lipofuscin. Retinosomes are lipid droplet containing retinol, retinoic acid, and retinol ester which are also sources of autofluorescence^31^,^32^. The lifetime fingerprint of pure retinol and retinoic acid with fluorescent lifetime shorter than LSS has been shown to be in a different spatial location of the lifetime phasor plot compared to the lipid droplet associated LLS reported here (See Supplementary Fig. 6)^12^. Furthermore, retinol and retinoic acid have a very prominent Raman band in the 1590–1600cm^−1^ range that was not observed in our Raman spectra^33^. The phasor distribution of pure retinol in Bovine serum albumin (BSA), excited at 740nm (2PE), is also shown in Supplementary Fig. 6.

ROS and oxidative stress are related to a myriad of pathological conditions including diabetes mellitus, obesity, inflammation, cancer, cardiovascular diseases, lung diseases and neurodegenerative diseases^4^,^5^,^6^,^7^,^8^,^9^,^10^ . Its role in pathogenesis has made it an important candidate for research on disease development, diagnosis and treatment routes. Thus a biomarker for oxidative stress could be used to elucidate pathways of disease development. Autofluorescence of oxidized lipid has the potential to be such a biomarker and in this work we show a unique detection approach by employing FLIM imaging.

Even though MPM still has limited application in clinical settings, it improves penetration for deep tissue imaging and in vivo animal models. Furthermore, as shown in this work, we can apply this imaging technique to live cells and freshly excised tissue.

The long lifetime species FLIM signature of oxidized lipids detected using the phasor approach is a promising, non-invasive tool to detect oxidative stress in biological systems. As far as we know, this is the first time a label-free fluorescent technique has been proposed for this purpose. As shown in this work, phasor analysis of FLIM allows an efficient way to uniquely identify intrinsic, autofluorescent marker of oxidative stress in cell cultures as well as tissue samples.

## Methods

### Instruments

Fluorescence lifetime imaging measurements of HeLa cells were performed on Zeiss LSM 710 microscope (Carl Zeiss, Jena, Germany) using a 40x water immersion objective, 1.2 N.A. (Carl Zeiss, Oberkochen, Germany). For the 2-Photon excitation laser source, Titanium:Sapphire MaiTai laser (Spectra-Physics, Mountain View, CA) was used with excitation at 740 nm. Image scan speed was 25.21 µs/pixel and image size is 256 × 256 pixels. For separating excitation from emission signal a dichroic at 690 nm was employed. The emission filter used was a bandpass 460/80 nm and photomultiplier tube (H7422P-40, Hamamatsu, Japan) was used for detection. FLIM data was acquired using A320 FastFLIM FLIMbox (ISS, Champaign, IL).

FLIM and third harmonic generation imaging of tissue sample were acquired using a custom- built upright deep tissue imaging microscope. The operation principle has been discussed previously^34^. For FLIM measurements, tissue sample was excited at 740 nm and emission filter employed was bandpass 405–590 nm. For THG, excitation of 1038 nm was used and signal was collected with bandpass filter 320–390 nm. Both FLIM and THG signals were collected in transmission geometry on the same sample.

For FLIM and THG data acquisition and processing, the SimFCS software developed at the Laboratory of Fluorescence Dynamics (LFD, UC Irvine) was used.

Coherent anti-Stokes Raman scattering (CARS) images were obtained by combining a 1064 nm, 76 MHz mode-locked Nd:Vanadate laser (Picotrain, High-Q, Hohenems, Austria) and a 817 nm beam tuned from a MIRA 900 (Coherent, Santa Clara, California). The two beams were overlapped both temporally and spatially, and sent into a laser scanner (Fluoview 300, Olympus, Center Valley, PA), attached to an inverted microscope (IX71, Olympus). The combined beams were then focused through a 20 × 0.75 NA objective lens (UPlanSApo, Olympus) onto the sample. The CARS signal was collected through the transmission channel by a photomultiplier tube (Hamamatsu, Japan) after passing through a 625/50 filter.

Spontaneous Raman spectra from the lipid droplets present in the cells were acquired with a commercial Raman microscope (InVia Confocal; Renisahw, Wotton-under-Edge, Gloucestershire, UK). The excitation wavelength at 523 nm was focused into the sample with a 50 × objective, and the scattered light was sent into the spectrometer that contained a 2400 l/mm grating. The autofluorescent lipid droplets are identified based on morphology. The Raman spectrum is then taken with 10s integration time, and the baseline was estimated by minimizing a non-quadratic cost function.

### Samples

HeLa cells were grown in Dulbecco's Modified Eagle Medium (D-MEM) (1X), liquid (high glucose) supplemented with 10% Fetal Bovine Serum, and 1% penicillin streptomycin (100I U/ml) at 37°C in a 5% CO incubator. For oleic acid treatment, the cells were cultured in 5% lipoprotein deficient serum, LPDS (Intracel, Frederick, Maryland) and 95% D-MEM for 24 hours. Fatty acid free bovine serum albumin was prepared by dissolving BSA powder (Sigma-Aldrich, St. Louis, Missouri) in 5% LPDS media. 400 µM oleic acid was prepared as a complex with BSA (OA/BSA) at molar ratio of 2:1. Cells were treated with OA/BSA complex overnight. For controls, three different dishes of HeLa cells were cultured in normal media and 5% LPDS media. For imaging, the cells were plated in glass bottom dishes (Matek Corporation, Ashland, Massachusetts). Prior to FLIM imaging, the oleic acid fed cells were washed with 1X Dulbecco’s Phosphate Buffered Saline, DPBS (Sigma-Aldrich). For CARS imaging, media was replaced with DPBS. 4% Paraformaldehyde (Sigma-Aldrich) solution was prepared to fix the cells for Raman spectroscopy measurements.

White adipose tissue was obtained from 5 month old adult female mice. Approximately 3mm diameter portions of fat from perigonadal and flank white adipose tissue depots were freshly excised from the mice and subsequently embedded in 1% low melt agarose in HBSS heated to 37°C between coverslips separated by 0.2 mm spacers. All imaging were strictly performed within 1 hour of tissue extraction. All animal procedures were performed with strict adherence to NIH OLAW and institutional IACUC guidelines.

For pure free and protein bound NADH FLIM measurements, 2.5 µM NADH were diluted in 10 mM NaH_2_PO_4_*H_2_O at pH = 7.4. For bound, it was mixed with 0.75 U/ml Lactate dehydrogenase (LDH). For lifetime measurement of retinoid in bovine serum albumin (BSA), retinol (Sigma-Aldrich) was dissolved in dimethyl sulfoxide, DMSO (EMD Millipore, California) and added to BSA (Sigma-Aldrich) in buffer.

## Additional Information

**Ethic Statement**: The experimental protocols were carried out in accordance with the Guide for the Care and Use of Laboratory Animals (NIH-OLAW) and were approved by the Institutional Animal Care and Use Committee at the University of California, Irvine (IACUC-2011-2978).

**How to cite this article**: Datta, R., Alfonso-Garcı´a, A., Cinco, R. & Gratton, E. Fluorescence lifetime imaging of endogenous biomarkerof oxidative stress. *Sci. Rep.* **5**, 9848; doi: 10.1038/srep09848 (2015).

## Supplementary Material

Supplementary Information Supplementary Information
