# Supplementary material for: Fluorescence lifetime imaging of endogenous biomarker of oxidative stress
Source: Sci Rep. 2015 May 20;5:9848. doi: 10.1038/srep09848 (PMC4438616; doi:10.1038/srep09848)
Supplement: Supplementary Information [file srep09848-s1.pdf]

Supplementary

Fluorescence lifetime imaging of endogenous biomarker of oxidative stress

Rupsa Datta[1], Alba Alfonso-García[2], Rachel Cinco[3], Enrico Gratton[1]*

1. Laboratory of Fluorescence Dynamics, Department of Biomedical Engineering, University of California, Irvine,

2. Department of Biomedical Engineering, University of California, Irvine

3. Department of Developmental & Cell Biology, University of California, Irvine

*Corresponding author e-mail: egratton22@gmail.com

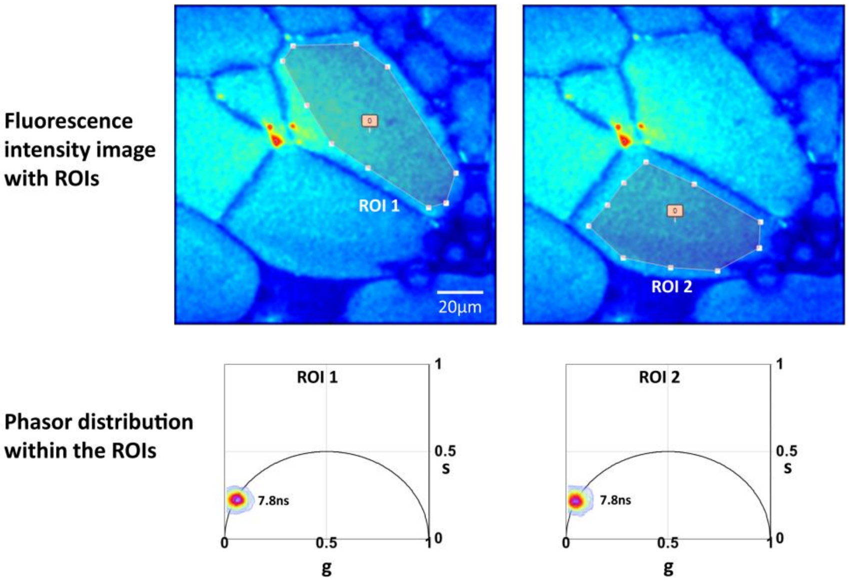

Supplementary Figure S1. Unique long lifetime distribution falls on universal circle

Images on top row are average fluorescence intensity image with region of interest (ROI) mask drawn manually choosing two different adipocyte within the imaged region. The corresponding phasor distributions of the ROIs are shown in the phasor plot below each image. The lifetime distribution and THG from this sample was shown in Fig. 1

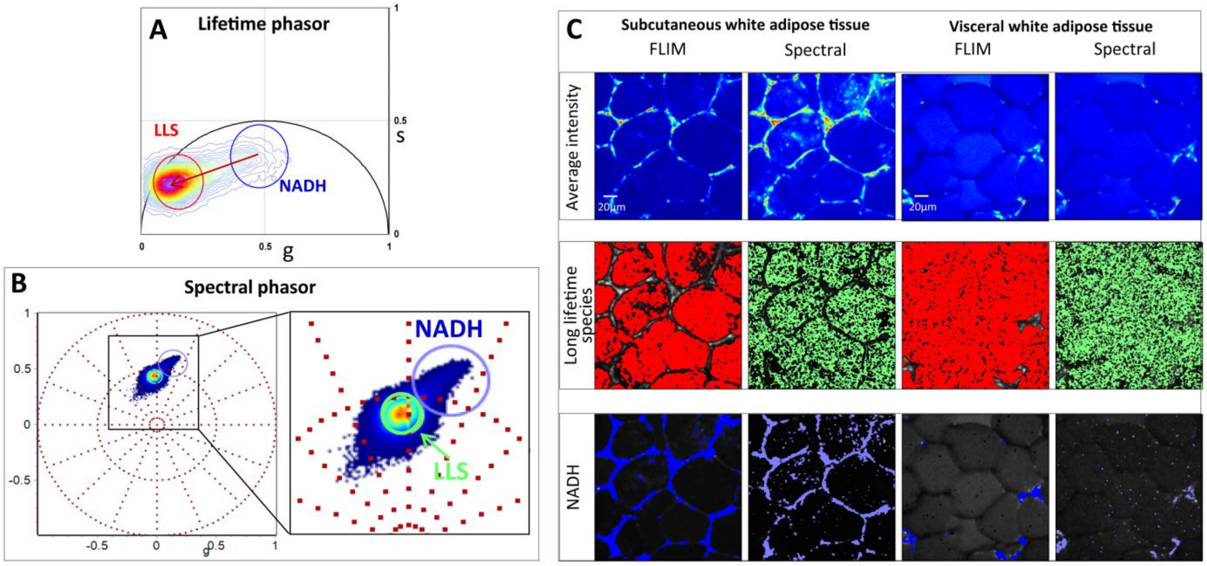

Supplementary figure S2. FLIM and spectral phasor analysis of visceral and subcutaneous WAT

A. FLIM phasor distribution of subcutaneous white adipose tissue and visceral white adipose tissue from the same mouse. Two separate lifetime population have been selected with red cursor (long lifetime species) and blue cursor (NADH). B. Left panel is the corresponding spectral phasor. Right panel is zoomed in spectral phasor distribution where two populations corresponding to long lifetime species and NADH are selected with green and purple cursors respectively. C. Top panel are average intensity images of white adipose tissue and visceral white adipose tissue in FLIM and spectral mode. Middle panel is the corresponding long lifetime FLIM and spectral phasor maps according to the masks selected in FLIM (A) and spectral phasor (B). Bottom panel is the NADH FLIM and spectral phasor maps according to the masks selected in FLIM (A) and spectral phasor (B).

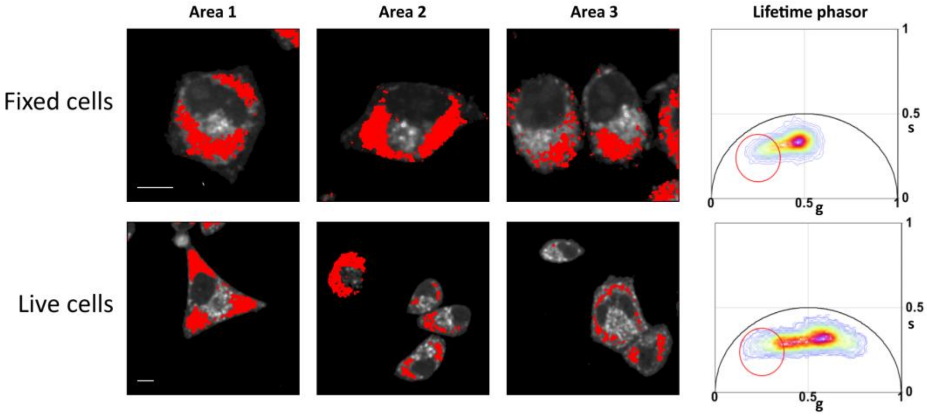

Supplementary figure S3. Comparison of LLS lifetime distribution in fixed and live HeLa cells

Top row first three images are LLS FLIM map (selected by red cursor in top row last panel) in fixed oleic acid treated HeLa cells. Top row last panel is the phasor distribution of the fixed samples.

Bottom row first three images are LLS FLIM map (selected by red cursor in bottom row last panel) in live oleic acid treated HeLa cells. Bottom row last panel is the phasor distribution of the live samples. The positions of the red cursors have been kept same in both the phasor plots. Fixed cell, Area 2 was shown in Fig. 6 along with its THG signal.

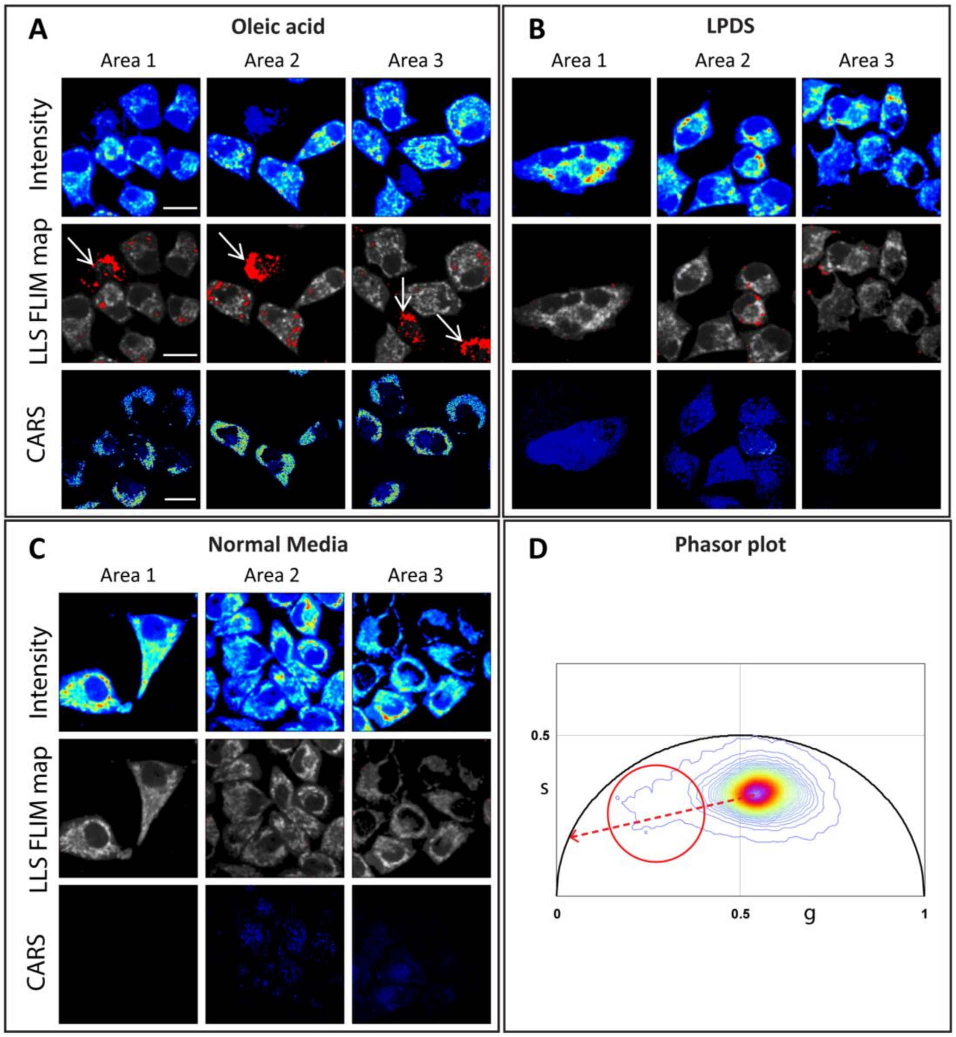

Supplementary Figure S4. FLIM and CARS imaging of HeLa cells

A Oleic acid treated HeLa cells. B. HeLa cells in LPDS media. C. HeLa cells in normal media. Top row are average fluorescence intensity images of three areas of 400μM oleic acid treated HeLa cells. Middle row are corresponding LLS FLIM maps. The red regions are pixels that have lifetime within the red cursor in the phasor plot in (D). Bottom row are normalized CARS images of the corresponding areas. Note, cells marked with white arrow in the middle panel of A are not present in the corresponding CARS images. Scale bar (20 μm) of the first image applies to all the images in that row. D. Lifetime phasor distribution of all the groups with red cursor selecting the LLS distribution.

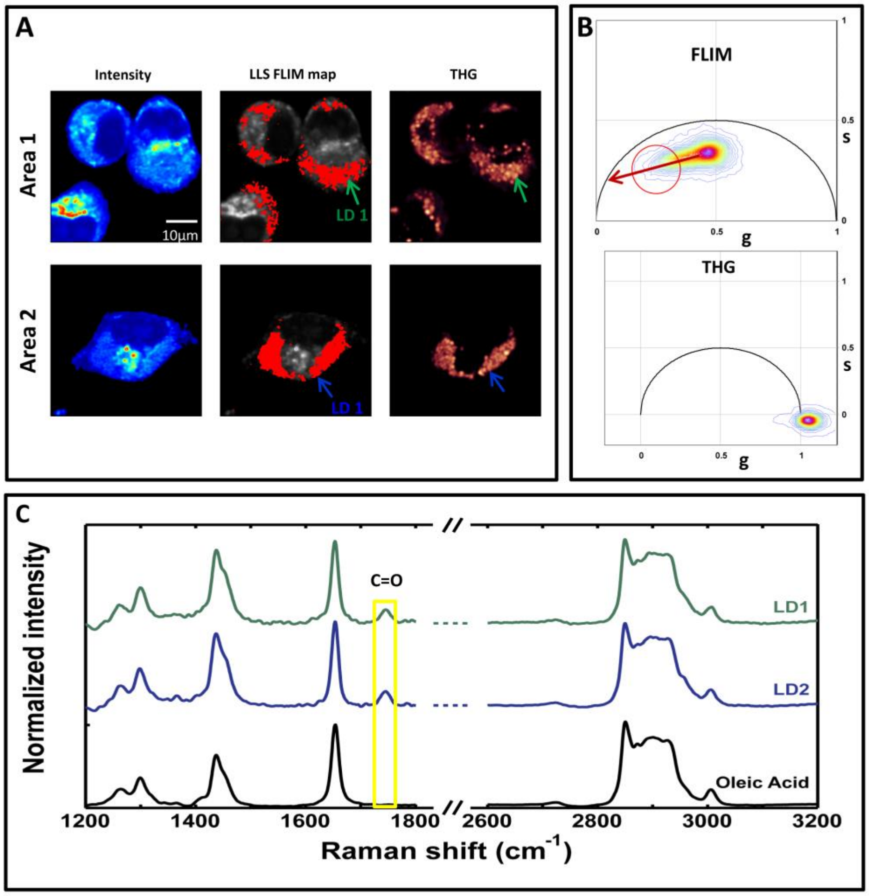

Supplementary Figure S5. FLIM, THG and Raman spectroscopy of LLS

A. Fluorescent intensity image (right column), FLIM map with LLS pixels in red (middle column) and THG image (left column) of oleic acid treated fixed HeLa cells. B. Top panel is the phasor distribution from the 2 areas shown in A. Red cursor selects the LLS distribution. Red dotted line is the oxidative stress axis. Bottom panel is the THG phasor distribution. C. Raman spectra of LLS containing lipid droplets in Area 1 (green curve) and Area 2 (blue curve) and pure oleic acid (black curve). The region from where Raman spectra were acquired is marked with an arrow of the corresponding Raman spectra curve color in B (green for Area 1, blue for Area 2) Yellow dotted box highlights the additional peak observed in the Raman spectra from biological sample which is not a feature of oleic acid. Note that Area 2 has been previously shown in Fig 6 to compare LLS with THG signal and its LLS lifetime distribution was matched with that of live HeLa cells in Supplementary Fig. 3 (Area 2)

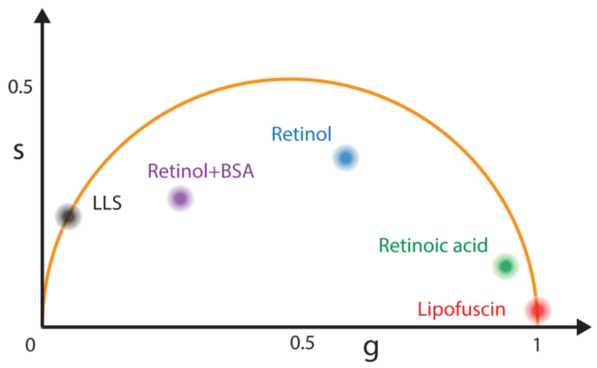

Supplementary Figure S6. FLIM phasor signature of autofluorescence related to lipid droplets

Schematic representation of FLIM phasor signatures of retinol (blue), retinoic acid (green), lipofuscin (red) retinol with BSA (purple) and LLS (black) to demonstrate the respective positions on the phasor plot.
